# A mutation in *TGFB3* associated with a syndrome of low muscle mass, growth retardation, distal arthrogryposis and clinical features overlapping with marfan and loeys–dietz syndrome

**DOI:** 10.1002/ajmg.a.36056

**Published:** 2013-07-03

**Authors:** Hugh Young Rienhoff, Chang-Yeol Yeo, Rachel Morissette, Irina Khrebtukova, Jonathan Melnick, Shujun Luo, Nan Leng, Yeon-Jin Kim, Gary Schroth, John Westwick, Hannes Vogel, Nazli McDonnell, Judith G Hall, Malcolm Whitman

**Affiliations:** 1 Children's Hospital Oakland Research InstituteOakland, California; 2 Department of Life Sciences, Ewha Women's UniversitySeoul, Republic of Korea; 3 National Institute on Aging, NIHBaltimore, Maryland; 4 Illumina, Inc.Hayward, California; 5 Odyssey Thera, Inc.San Ramon, California; 6 Department of Pathology, Stanford University Medical SchoolStanford, California; 7 Department of Medical Genetics, University of British ColumbiaVancouver, British Columbia, Canada; 8 Department of Developmental Biology, Harvard School of Dental MedicineBoston, Massachusetts

**Keywords:** transforming growth factor beta, Marfan syndrome, Loeys–Dietz syndrome, distal arthrogryposis, low muscle mass, bifid uvula, exome sequencing, de novo mutation, hyomyoplasia

## Abstract

The transforming growth factor β (TGF-β) family of growth factors are key regulators of mammalian development and their dysregulation is implicated in human disease, notably, heritable vasculopathies including Marfan (MFS, OMIM #154700) and Loeys–Dietz syndromes (LDS, OMIM #609192). We described a syndrome presenting at birth with distal arthrogryposis, hypotonia, bifid uvula, a failure of normal post-natal muscle development but no evidence of vascular disease; some of these features overlap with MFS and LDS. A de novo mutation in TGFB3 was identified by exome sequencing. Several lines of evidence indicate the mutation is hypomorphic suggesting that decreased TGF-β signaling from a loss of TGFB3 activity is likely responsible for the clinical phenotype. This is the first example of a mutation in the coding portion of TGFB3 implicated in a clinical syndrome suggesting TGFB3 is essential for both human palatogenesis and normal muscle growth.

## INTRODUCTION

The transforming growth factor β (TGFB) family of growth factors are key regulators of mammalian development and their dysregulation is implicated in human disease, notably, heritable vasculopathies including Marfan (MFS, OMIM #154700) and Loeys–Dietz syndrome (LDS, OMIM #609192). We describe a syndrome presenting at birth with distal arthrogryposis, hypotonia, bifid uvula, a failure of normal post-natal muscle development without evidence of vascular disease; some features overlap with MFS and LDS. A de novo mutation in *TGFB3* was identified by exome sequencing. Several lines of evidence indicate the mutation is hypomorphic, suggesting that decreased TGFB signaling from a loss of TGFB3 activity is likely responsible for the clinical phenotype. This is the first example of a mutation in the coding portion of *TGFB3* implicated in a clinical syndrome, suggesting TGFB3 is essential for both human palatogenesis and normal muscle growth.

## CLINICAL REPORT

The proband is a 9-year-old European-American female born to nonconsanguineous, healthy parents with two older healthy children and no previous miscarriages. Mother was 34 years old and father was 42 at delivery. No cytogenetic abnormality was observed on chromosome samples obtained at 19 weeks gestation by amniocentesis. The pregnancy was unremarkable and the mother reported that the fetus moved in utero similarly to older siblings. The child was born at full term by repeat caesarian; Apgars were 9 and 9 at 1 and 5 min, respectively. The birth weight was 2.9 kg (5th centile), length was 51 cm (50th centile), and OFC was 35.75 (>50th centile). The physical exam showed contractures in the right hand, most severe in the 3rd and 4th fingers (see Fig. S1 in Supplementary online material) and all toes. The digits on the left hand and foot showed minimal contractures. Range of motion of knees, hips, elbows, jaws, and back were considered normal. Hands and feet appeared long and narrow although no measurements were taken. There was a midline facial nevus flammeus and mild hypotonia.

**Figure 1:**
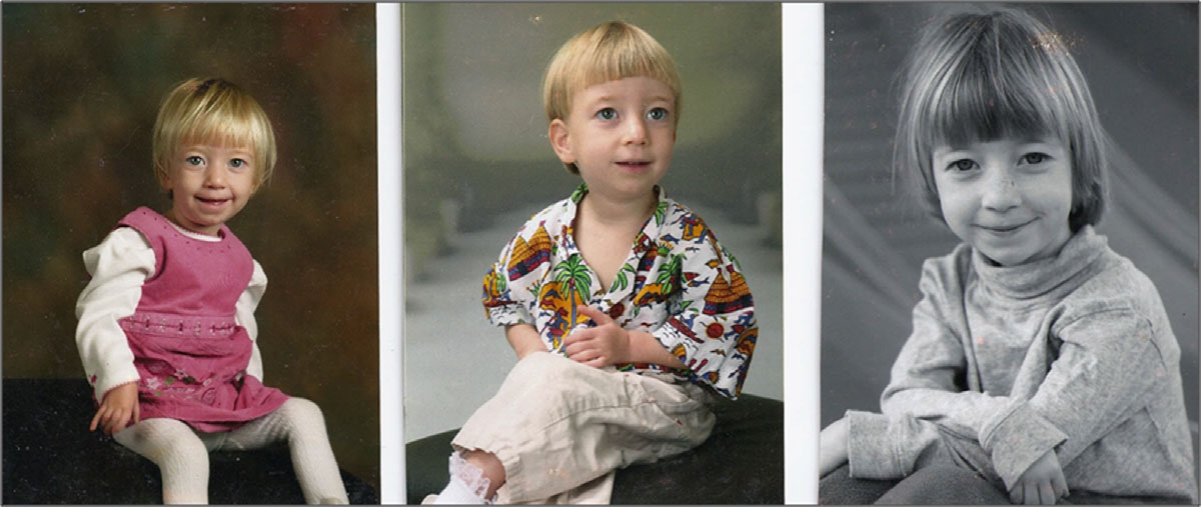
Pictures of the proband at ages 17 months, 36 months, and 6 years. Evident at age 17 months are the prominent eyes, hypertelorism, tubular nose, and retrognathia. Evident at age 36 months are a normal ear, retrognathia, and less pronounced hypertelorism. At age 6 years the inner canthal distance was within normal limits.

At age 3 months, the patient was evaluated for failure-to-thrive because of low weight (4.2 kg <5th centile) and a delay in gross motor function. An evaluation at 17 months showed that she did not crawl or roll, but could stand with support. She weighed 7.5 kg (<1st centile) and was 76 cm in height (5th centile) (Fig. 1, left picture). Also noted was bilateral pes planus, mild pectus excavatum, hyperextensibility of multiple large joints, and mild retrognathia. Eyes appeared prominent and hypertelorism was present with an outer canthal distance of 7.8 cm and an inner canthal distance of 2.8 cm (>97th centile). Her skin had normal texture, tension, and wound healing. Head circumference was 45 cm (25th centile) with normally placed and well-formed ears. A bifid uvula with intact hard palate, normal arch, and normal voice quality were present. A small metopic ridge and normal teeth were observed. The sclerae were blue. She had marked contractures at the proximal phalangeal joints of the right second and third digits and toes bilaterally (R > L). Her motor examination revealed decreased bulk in all appendicular and axial muscles, strength 1/5, low tone, and diminished reflexes throughout. Markedly reduced subcutaneous fat was noted.

The patient could stand independently with a positive Gower sign at 20 months, and walked at 24 months in a “hip waddle” fashion. Her neurocognitive development was appropriate for age. Slit lamp examination of both eyes was normal. Radiographic studies of the hands and pelvis showed age-appropriate ossification and bilateral coxa valga deformity, respectively.

At age 3 years, a 3-year trial of losartan at a dose of up to 2.0 mg/kg/day produced no change in muscle strength or mass (Figs. 1, middle picture and 2). At the age seven, she weighed 15.5 kg (<1st centile) and was 115 cm (5th centile) in height; the physical exam was otherwise unchanged. The skin had normal texture, subcutaneous fat was minimal, and marked hyperextensibility was present in elbows and knees. No abnormal spinal curvature was evident. A right quadricep muscle biopsy taken at age 7 years showed normal a checkerboard pattern with Type 1 fiber predominance but mild and focal Type 1 fiber disproportion consistent with disuse or decreased usage (type 1: 47 µ vs. Type 2: 50 µ) (see Figs. S4–S7 in Supplementary online material).

**Figure 2:**
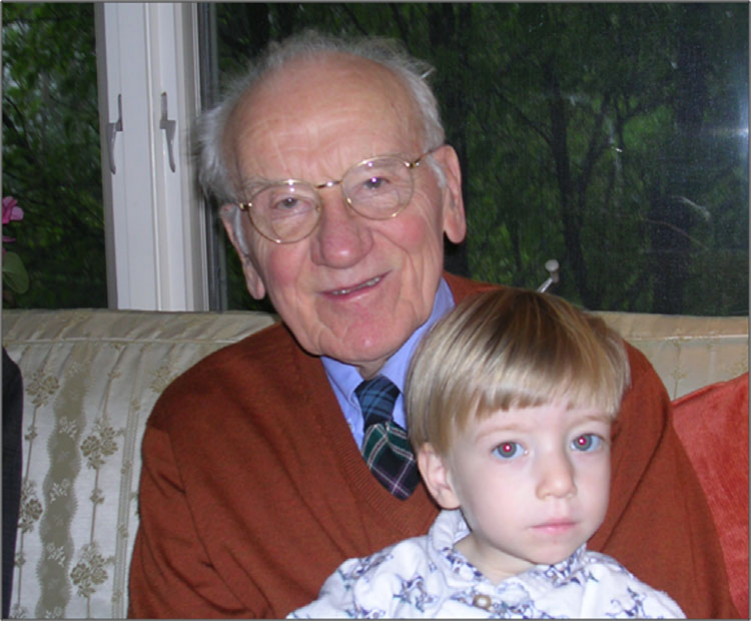
Picture of the proband at age 30 months with V.A. McKusick, M.D. Evident in the proband are the blue sclera, the well-placed and normal ear, tubular nose with metopic ridge, mild hyperterlorism, retrognathia, and hypomalar eminences.

Yearly echocardiograms beginning at 18 months showed no cardiac defect or dysfunction. An echocardiographic examination at the age 6.5 years measured the aortic annulus and aortic root at 1.34 cm (z score 0.59) and 1.72 cm (z score 0.39), respectively; the pulmonary artery dimensions were also consistently within the normal range. Visual acuity remained normal. The proband had physical and occupational therapy since the age 6 months, and has worn orthotic foot appliances since age 2. Although digit contractures persist, at age 9 she has very functional hands.

## METHODS

Genomic DNA was extracted from peripheral mononuclear blood cells from the proband, two unaffected sibs, and parents. The details of the exome and Sanger sequencing and the Xenopus and cultured cell methods are described in Supplementary online material. The focus of the sequence data filtering was for novel non-synonymous variants that were unique. These included heterozygous novel missense and nonsense substitutions and frame-shifting insertions and deletions (in/dels) not detected in other family members. In addition, we looked for homozygous non-synonymous variants (missense or nonsense substitutions or frame-shifting in/dels) where she was the only family member homozygous for the damaging allele, and where both parents were heterozygous. Finally, we looked compound heterozygosity of deleterious alleles in genes associated with heritable disorders of connective tissue.

## RESULTS

The proband shared the clinical feature of low muscle mass with hypotonia with three related conditions: Marfan, Loeys–Dietz, and Beals–Hecht syndrome (BHS, OMIM # 121050). She was hyperteloric and had a bifid uvula, the two non-vascular findings that define LDS; the skeletal findings typical of MFS included arachnodactyly, pectus excavatum, pes planus, and hyperextensible large joints. The proposita did not meet the diagnostic criteria established for MFS, BHS or LDS. Among the inconsistencies with these diagnoses were significant growth retardation, the absence of cardiovascular findings, and the distinct muscle histopathology. These three syndromes are allied in their pathophysiology. TGF-β signaling is dysregulated in the first two conditions and analogous pathophysiology is suspected in BHS, given the similar functions of FBN1 and FBN2. The clinical overlap with these autosomal dominant syndromes and the unaffected status of the parents suggested a de novo mutation acting as a dominant trait in a gene affecting TGF-β signaling. Analysis of the six genes (*TGFB2*, *TGFBR1*, *TGFBR2*, *SMAD3*, *FBN1*, *FBN2*) associated with MFS, LDS, or BHS identified no mutation. We sought to identify de novo mutations through exome sequencing; consistent with expectations [Neale et al., 2012], we found two nucleotide changes unequivocally unique to the proband, in *CDH2* and *TGFB3*.

A nonsense mutation was found at codon 70 in *CDH2* (*c.208C*>*T*, *pQ70X*), encoding the 907 amino acid protein cadherin-2 or N-cadherin. Seventeen *CDH2* SNVs predicted to be damaging by PolyPhen are reported in the NHLBI exome variant server (EVS) database. In dermal fibroblasts isolated from the proband, the concentration of N-cadherin by Western blot was not statistically different from six age-matched, passage-matched controls (see Supplementary online material, Fig. S8). A second de novo mutation was found in *TGFB3* (c.1226G>A; pC409Y). Validation by Sanger sequencing confirmed that this mutation was de novo in the proband. No ligand-coding sequence variants have been reported in *TGFB3* including dbSNP130, EVS, or the 1000 Genomes dataset.

**Figure 3:**
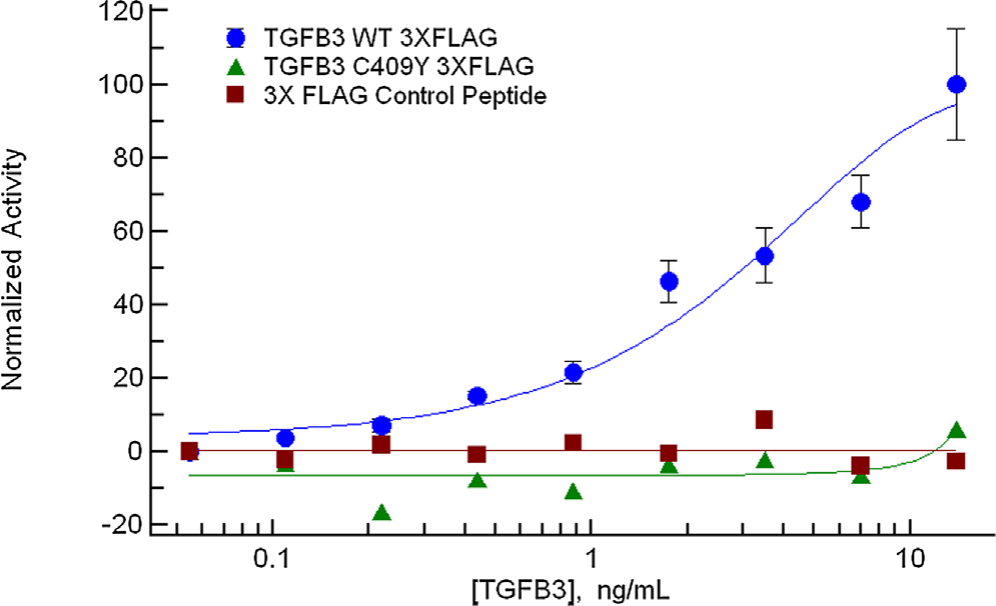
TGFB3^C409Y^ does not activate TGF-β signaling. Constructs containing FLAG-tagged *TGFB3^WT^* and *TGFB3^C409Y^* were expressed in HEK293T cells and secreted FLAG-tagged proteins were immunoprecipitated under native conditions and normalized to anti-FLAG reactivity. Serial dilutions of these proteins were applied to HEK293T cells transfected with p3TP-Lux (TGF-β reporter) and pRL-CMV (normalizer). Error bars are standard error of the mean (N = 3).

To determine the effect of the cysteine-to-tyrosine substitution on TGFB3 function, we co-transfected an epitope-tagged wild-type (w-t) *TGFB3* cDNA or a similarly tagged *TGFB3*^*G1226A*^ cDNA under the control of a CMV promoter into 293T cells (see Supplementary online material). Conditioned medium (CM) was collected, the tagged TGFB3 species purified by immunoprecipitation and the relative TGF-β signaling activities compared using HeLa cells transfected with the reporter plasmid, p3TPLux, a Smad2-responsive reporter. Figure 3 shows that the wild-type (w-t) *TGFB3* gene generated a transcriptional TGF-β signal, whereas the *TGFB3*^*G1226A*^ gene construct did not. We conclude that the mutant allele encodes a TGFB3 ligand that is not functional.

**Figure 4:**
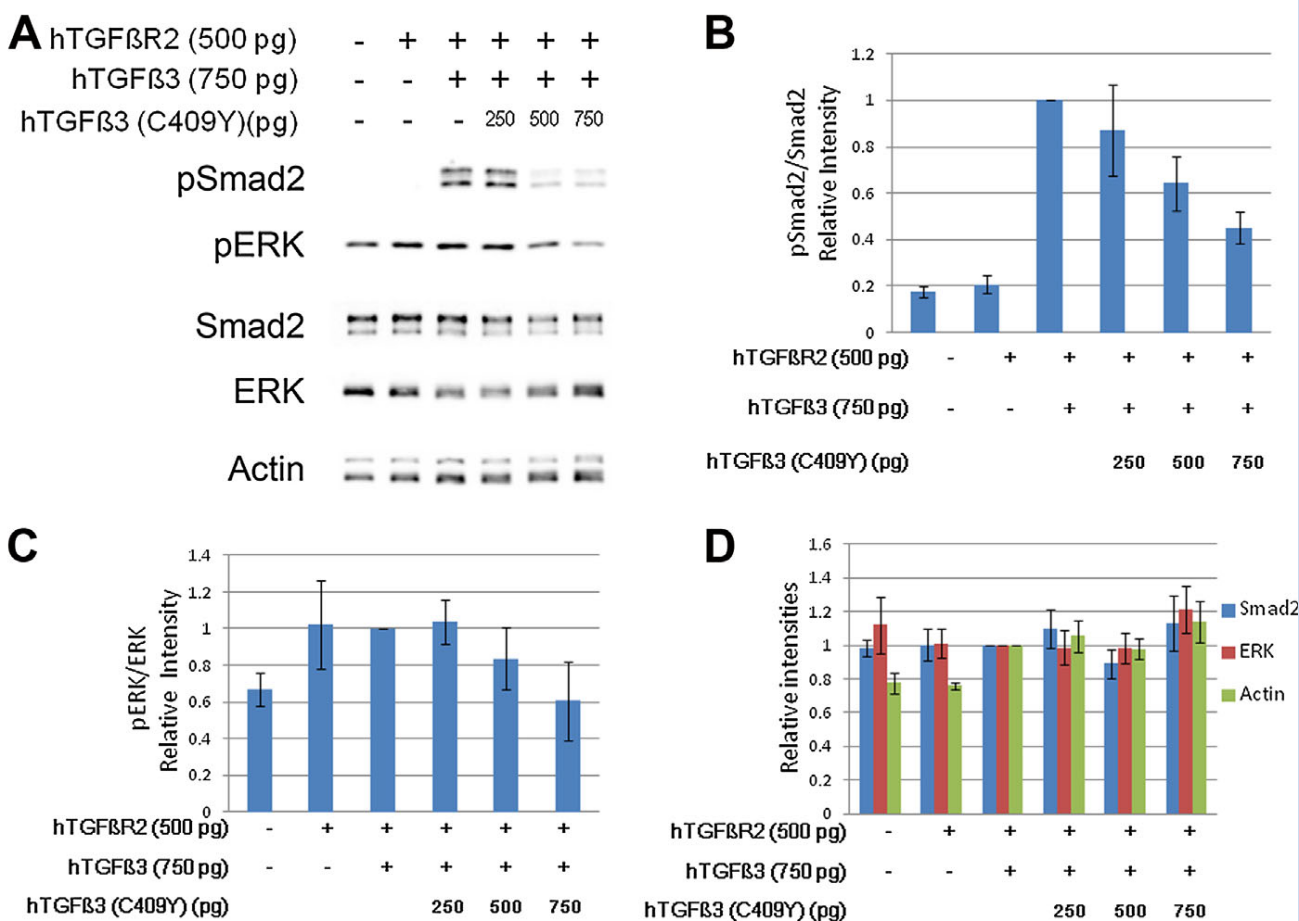
TGFB3^C409Y^ acts as a dominant inhibitor of TGF-β signaling. Indicated amount of synthetic mRNAs were microinjected into Xenopus embryos after fertilization, and embryos harvested at Stage 9 for Western blot analysis. A representative Western blot is shown in A. B–D: Quantitation of densitometric analysis of three independent experiments is shown, measuring phosphorylated SMAD (pSMAD2) activation (pSMAD2/total SMAD2 ratio), pERK activation (pERK/ERK ratio), and total SMAD2, ERK, and cytoplasmic actin levels, respectively, following injection of mRNAs as indicated. Data are presented with error bars representing standard error of the mean. For each experiment, the intensity Western signal was standardized relative to the wild type *TGFB/TGFBR2* co-injected condition (lane 3 of panels B–D).

**Figure 5:**
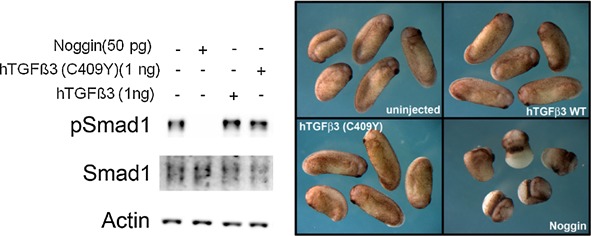
TGFB3^C409Y^ does not disrupt BMP signaling during *Xenopus* gastrulation. Indicated amounts of synthetic mRNAs for human *TGFB3*, *TGFB3^C409Y^* and *Xenopus* Noggin were injected into *Xenopus laevis* embryos to assay perturbations of BMP signaling during gastrulation. The levels of C-terminal phosphorylated SMAD1 (pSMAD1) were examined by immunoblotting. SMAD1 and actin were used as loading controls. Pictured in the lower panel are intact embryos; the BMPR antagonist Noggin disrupts gastrulation while TGFB3^C409Y^ does not.

Xenopus embryos provide a sensitive assay system for assessing effects of ectopically expressed proteins on TGF-β (SMAD2 and ERK1/2) and BMP (SMAD1) signaling. To examine how *TGFB3*^*G1226A*^ altered TGF-β signaling in Xenopus embryos, RNA from the w-t human *TGFB3* allele and the *TGFB3*^*G1226A*^ allele were injected into fertilized Xenopus eggs either separately or together using a constant amount of RNA from the w-t allele with an increasing amount of RNA from the mutant allele. Prospective ectodermal tissue from late blastula embryo was examined by Western blot for phosphorylation of SMAD2 and ERK1/2 (pSMAD2 and pERK1/2, respectively). Figure 4 shows that RNA from the w-t human *TGFB3* allele results in a significant increase in pSMAD2 and pERK1/2 signals by Western blot as expected (see Supplementary online material Fig. S9 for controls). The TGFB3-dependent pSMAD2 and pERK1/2 signals are significantly diminished, however, with increasing amounts of the mutant RNA relative to wild-type. A 1:1 ratio of mutant-to-wild-type *TGFB3* RNA diminished the pSMAD2 and pERK1/2 signals to approximately 40% and 60%, respectively, of that generated by the w-t *TGFB3* RNA alone. Gastrula stage embryos require activation of SMAD1 by endogenous BMP2/4/7 for normal dorsal-ventral patterning. Ectopic expression of *TGFB3*^*G1226A*^ had no effect on either SMAD1 phosphorylation (pSMAD1) or dorsal ventral patterning in Xenopus gastrulae (Fig. 5). We conclude that in the frog system the mutant *TGFB3*^*G1226A*^ allele, when co-expressed with w-t *TGFB3*, has a dominant negative effect on TGF-β signaling measured by pSMAD2 and pERK1/2, but does not affect signaling by BMP2/4/7 ligands measured by pSMAD1.

## DISCUSSION

The principal clinical concerns for this patient have been growth retardation, weakness related to decreased muscle mass and uncertainty about her risk for vascular disease. Distal arthrogryposis indicative of reduced fetal movement suggested an inborn error of development affecting muscle mass and other developing mesenchymal tissues, including the soft palate.

A muscle biopsy showed essentially normal fiber size and architecture. There was no evidence of chronic dystrophic or inflammatory changes presenting a strikingly different histologic picture compared to the myopathic findings in congenital or classical MFS in which endomysial thickening, fat deposition, split fibers, fibrosis, and marked fiber size disproportion are consistent histopathological observations. The phenotypic discordance in the proband—significant underdevelopment of muscle mass or hypomyoplasia with the absence of abnormal cardiac or aortic findings by age 8 years—suggests a pathophysiology distinct from that caused by excess TFG-β signaling observed with *FBN1* mutations in MFS. Furthermore, in one mouse model of MFS, muscle mass was normal and there was complete histologic restoration of muscle architecture after treatment with pan-anti-TGFB antibodies or losartan, suggesting that excess TGF-β signaling in MFS has its major pathological effects on muscle during the post-natal growth phase of muscle development [Cohn et al., 2007]. The proband had no change in strength or muscle mass after a significant therapeutic trial of losartan, further suggesting a pathophysiology distinct from mutations enhancing TGF-β signaling.

The proband was heterozygous for two de novo mutations. The CDH2 levels were not statistically significantly different from control fibroblasts (see Supplementary online material). Mice heterozygous for a null mutation in *Cdh2* were phenotypically normal at 2 years; specifically, muscle mass was not affected [Garcia-Castro et al., 2000]. We conclude that the proband's mutation in *CDH2* (c.208C>T) is unlikely to be driving the clinical phenotype, though we cannot exclude some modifying effects.

The second de novo mutation was *TGFB3*^*G1226A*^, a novel variant occurring in the last exon of *TGFB3*. The mature TGFB3 ligand is a homodimer of the C-terminal 112 amino acids of the *TGFB3* mRNA translation product [Derynck and Miyazono, 2008]. The mutation, *TGFB3*^*G1226A*^, substitutes a tyrosine for a cysteine (TGFB3^C409Y^). This penultimate cysteine is conserved in all TGFB family member ligands including orthologues in *Caenorhabditis elegans* and *Drosophila melanogaster* defining a structure called the “cysteine knot”, a conformation essential for normal ligand activity [Daopin et al., 1992; Mittl et al., 1996]. Site-directed mutagenesis has established the essentiality of this cysteine in TGFB isoforms [Brunner et al., 1992].

The *TGFB3*^*G1226A*^ allele coded for a ligand with no apparent signaling activity (Fig. 3) and the mutant allele, when co-expressed in embryos with the w-t allele, reduced TGF-β signaling activity to approximately 40% (Fig. 4). In the presence of the w-t and mutant allele there was no apparent effect on BMP2/4/7 signaling (Fig. 5), which is essential for normal skeletal muscle development [Wang et al., 2010a–2010b]. We conclude that the loss of cys^409^ in TGFB3 destroys TGFB3 signaling activity and when the mutant ligand is present with the wild-type TGFB3 protein, TGFB signaling is reduced. Based on these biochemical data in the context of the clinical findings, we conclude that the *TGFB3* mutation most likely accounts for the clinical findings.

What mechanism might account for hypomyoplasia? One pathophysiologic explanation centers on the critical role TGF-β signaling plays at specific embryonic stages of early mesenchymal development, including myogenesis and palatogenesis involving an epithelial-to-mesenchymal transition (EMT) [Kalluir and Weinberg, 2009; Pelton et al., 1990–1991]. TGFB3 is essential for palatal confluence [Nawshad and Hay, 2003; Proetzel et al., 1995] and mutations in *TGFB*3 have been linked to cleft palate. TGF-β signaling is also required for normal myogenesis following formation of the paraxial somite, the source of skeletal muscle precursors for myogenesis in the trunk and limbs [Derynck et al., 2008]. TGFB3 is the most abundant TGFB isoform in developing skeletal muscle [Pelton et al., 1991]. The dorsal portion of the somites retains an epithelial structure called the dermomyotome which undergoes progressive EMT forming myogenic precursors that later generate embryonic skeletal muscle. Partial or incomplete EMT may diminish the number of embryonic muscle precursor cells, ultimately reducing post-natal myofibril mass but not affecting muscle architecture. Thus, a deficiency of TGFB3 during early myogenesis could result in hypomyoplasia throughout development that clinically mimics, but is etiologically and histologically distinct from, the myopathy caused by excess TGF-β signaling found in MFS. Given the role GDF8 (myostatin) might play in embryonic muscle development, we cannot exclude the possibility that reduced TGFB3 signaling via its cognate Type I receptor (TGFBR1or ALK5) allows GDF8 to signal through the same receptor relatively unopposed, effectively enhancing the negative effect myostatin has on muscle precursor cell expansion [Lee et al., 2004; Amthor et al., 2006; Manceau et al., 2008]. Similarly, given that TGFB3 is reported to induce SMAD1/5 as well as SMAD2/3 phosphorylation, we cannot exclude the possibility that diminished pSMAD1/5 signaling permits premature myocyte differentiation at the expense of expansion of the adult satellite cell population [Daly et al., 2008; Ono et al., 2011; Lee et al., 2012]. These possible mechanisms in which TGFB3 might influence muscle development and growth could be operative together; none are exclusive of the others.

In summary, we describe a clinical syndrome characterized by abnormal development of several mesenchymal-derived tissues including muscle and cranio–palato-facial structures accompanied by low muscle mass, growth retardation, distal arthrogryposis and other secondary changes. Though the proband shares some clinical features with known syndromes that enhance TGF-β signaling, such as MFS and LDS, her findings are clinically distinct [Holm et al., 2011]. We identified a mutation in *TGFB3* and demonstrate in model systems that the altered TGFB3 ligand results in a decrease in canonical and non-canonical TGF-β signaling, suggesting that the phenotype is a consequence of a hypomorphic allele. The full phenotypic spectrum and natural history of *TGFB3* mutations must await other cases; we can, however, assert from these observations that the development of normal muscle mass appears to require a minimum of TGFB3 signaling, as does complete palatal fusion. While loss-of-function mutations in the *TGFB2*–*TGFBR*–*SMAD3* axis appear to enhance TGFB1 or TGFB2 expression in vascular tissue with at times catastrophic consequences, mutations in other components of the pathway, such as *TGFB3* may not have the same effects on vascular tissue or developing muscle. Clearly, ligand and tissue-specific factors contribute to the distinct clinical findings that render each syndrome unique.

### URLs

Database of Genomic Variants (DGV), http://projects.tcag.ca/variation/

dbSNP, http://www.ncbi.nlm.nih.gov/SNP/

DECIPHER (Wellcome Trust Sanger Institute), http://decipher.sanger.ac.uk/

Exome Variant Server (NHLBI Exome Sequencing Project, Seattle,WA), http://evs.gs.washington.edu/EVS/

GenBank, http://www.ncbi.nlm.nih.gov/Genbank/

Human Genome Mutation Database (HGMD), http://www.hgmd.org/

NCBI Reference Sequence (RefSeq), http://www.ncbi.nlm.nih.gov/RefSeq/

Online Mendelian Inheritance in Man (OMIM), http://www.omim.org

http://www.openbioinformatics.org/annovar/

UCSC Genome Browser, http://genome.ucsc.edu/

1000 Genomes, ftp://ftp-trace.ncbi.nih.gov/1000genomes/ftp/

Ingenuity® Variant Analysis™ software (www.ingenuity.com/variants) was used in this study. An interactive online supplement is available at https://variants.ingenuity.com/Rienhoff2013 which provides direct access to the dataset discussed in this manuscript.

## Notes

FIG. S1. The hands of the proband, age 8.

FIG. S2. The lower extremities of the proband, age 8.

FIG. S3. The face of the proband, age 4.5. Notable are the blue sclera, hypertelorism, malar hypoplasia, and tubular nose.

FIG. S4. Right quadricep biopsy (40×) stained with hematoxylin and eosin showing normal muscle fiber architecture.

FIG. S5. Right quadricep biopsy (40×) treatedwithATPase,pH9.4, showing the size and distribution of myofibers. Type 1 fibers are light; Type 2 fibers are dark.

FIG. S6. Right quadricep biopsy (40×) with trichrome staining showing normal intramyofibril membranes and interstitial collagen.

FIG. S7. Distribution of size in Type 1 and Type 2 fibers in representative sections of the proband’s muscle.

FIG. S8. N-cadherin expression levels. Protein expression levels of N-cadherin in whole cell lysate from human skin fibroblasts analyzed by Western blot. b-Tubulin was used as a loading control. Values expressed are normalized to loading controls.

FIG. S9. TGFB3 signaling in Xenopus embryos. Indicated amount of synthetic mRNAs were microinjected into Xenopus embryos after fertilization, and embryos harvested at Stage 9 forWestern blot analysis. This representative Western blot is shown to indicate that any of the injected synthetic mRNAs are not sufficient along to trigger phosphorylation of Smad2. As seen in Figure in the text, human TGFB3 and the TGFBR2 are required together to trigger the phosphorylation of Smad2.
