# Supplementary figures and images for: A mutation in TGFB3 associated with a syndrome of low muscle mass, growth retardation, distal arthrogryposis and clinical features overlapping with marfan and loeys–dietz syndrome
Source: Am J Med Genet A. 2013 Jul 3;161(8):2040–6. doi: 10.1002/ajmg.a.36056 (PMC3885154; doi:10.1002/ajmg.a.36056)

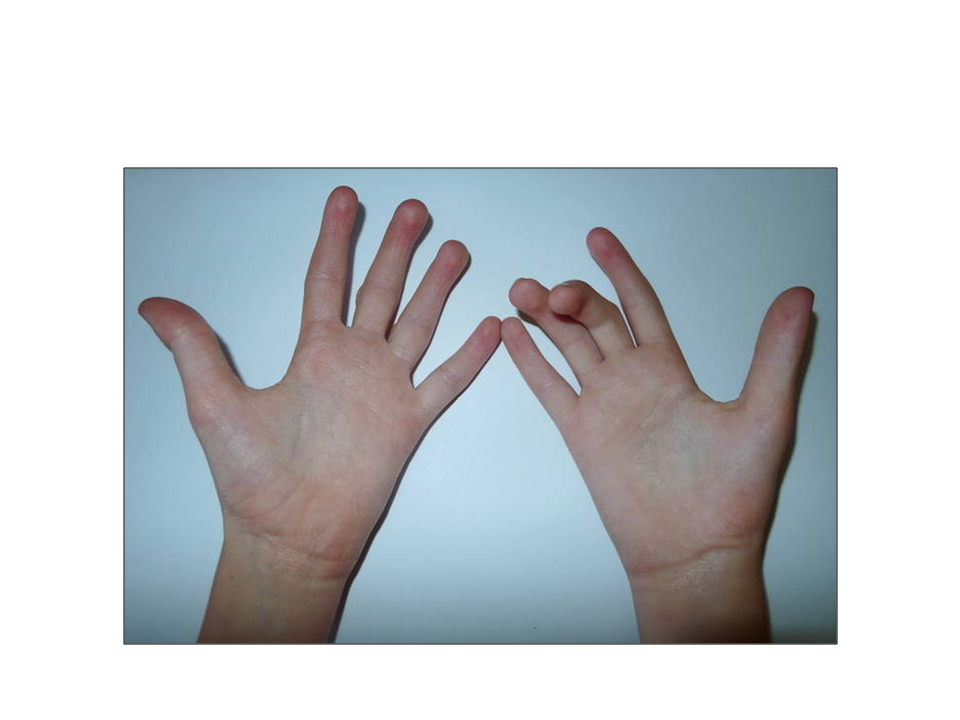

Supplement: Supplementary file 1 — FIG. S1. The hands of the proband, age 8. [file ajmg0161-2040-sd1.tif]

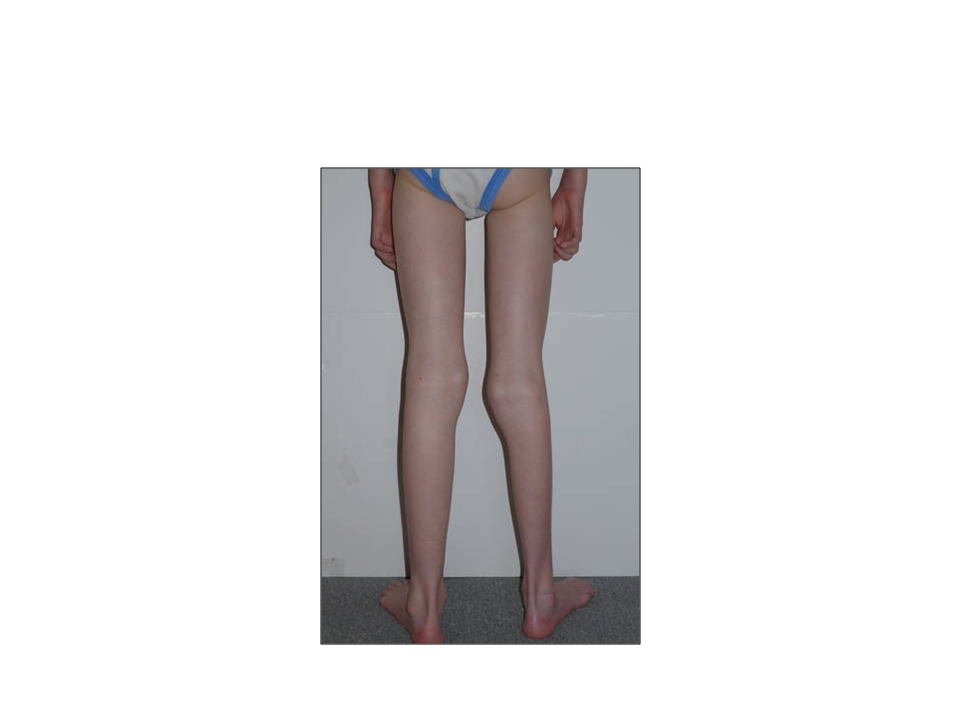

Supplement: Supplementary file 2 — FIG. S2. The lower extremities of the proband, age 8. [file ajmg0161-2040-sd2.tif]

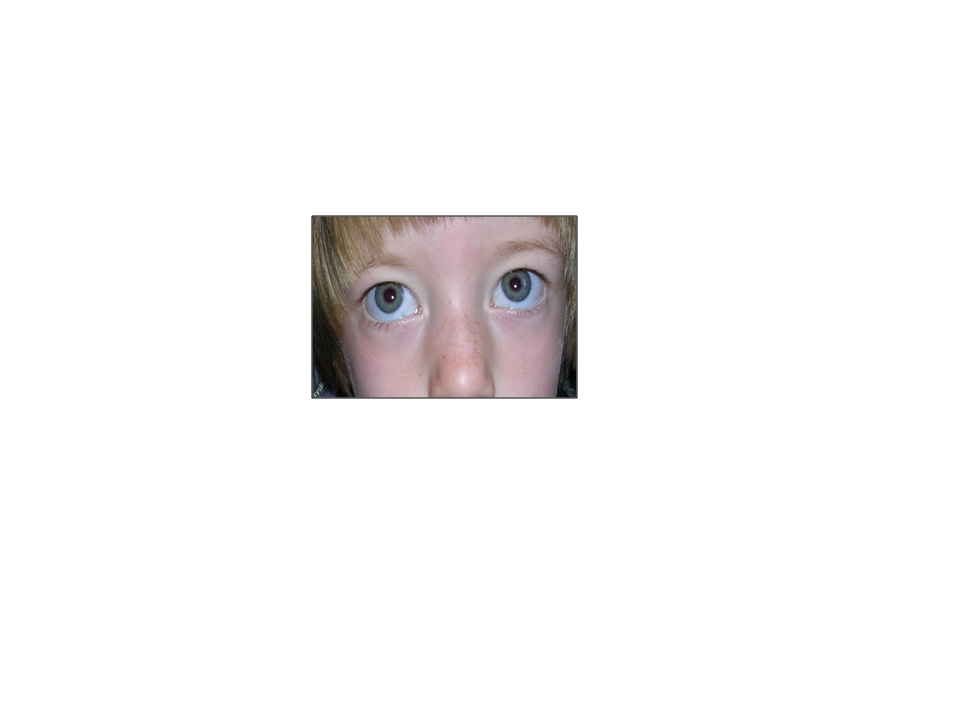

Supplement: Supplementary file 3 — FIG. S3. The face of the proband, age 4.5. Notable are the blue sclera, hypertelorism, malar hypoplasia, and tubular nose. [file ajmg0161-2040-sd3.tif]

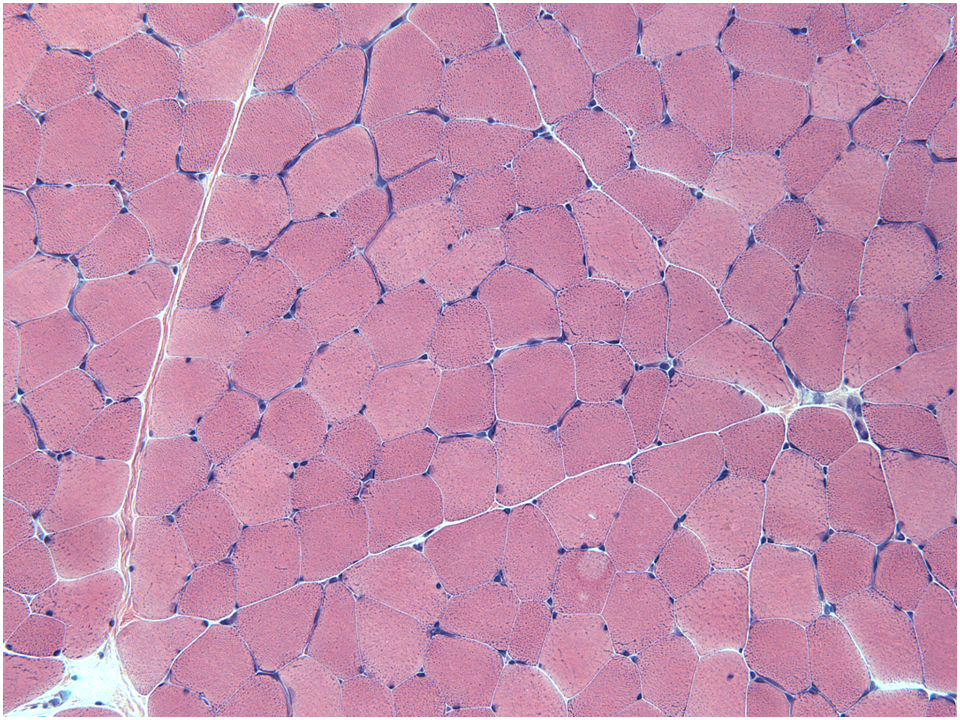

Supplement: Supplementary file 4 — FIG. S4. Right quadricep biopsy (40×) stained with hematoxylin and eosin showing normal muscle fiber architecture. [file ajmg0161-2040-sd4.tif]

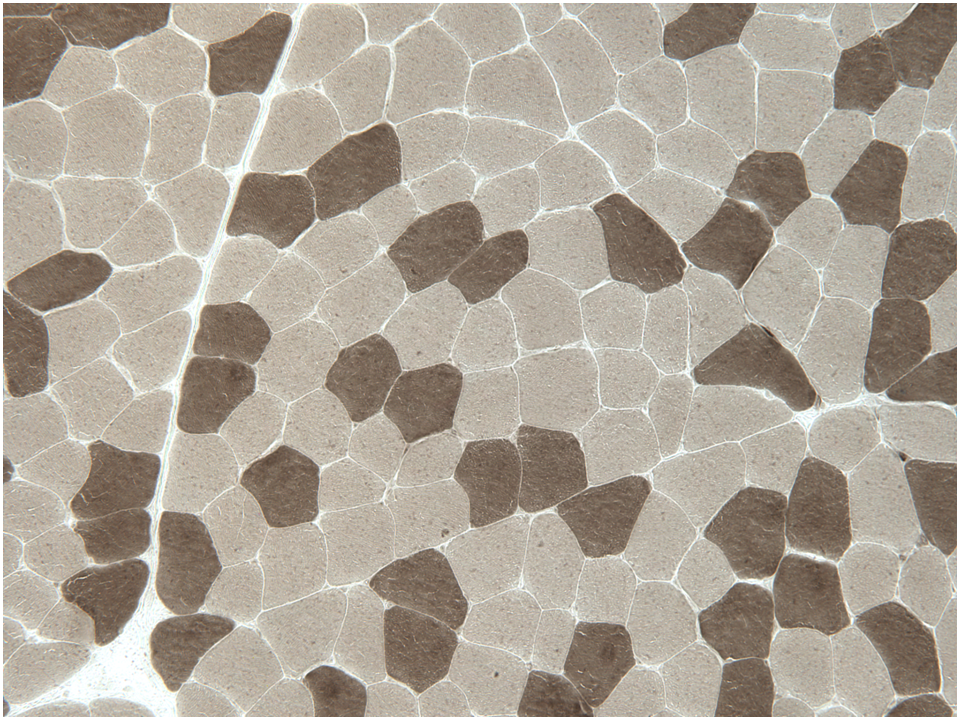

Supplement: Supplementary file 5 — FIG. S5. Right quadricep biopsy (40×) treatedwithATPase,pH9.4, showing the size and distribution of myofibers. Type 1 fibers are light; Type 2 fibers are dark. [file ajmg0161-2040-sd5.tif]

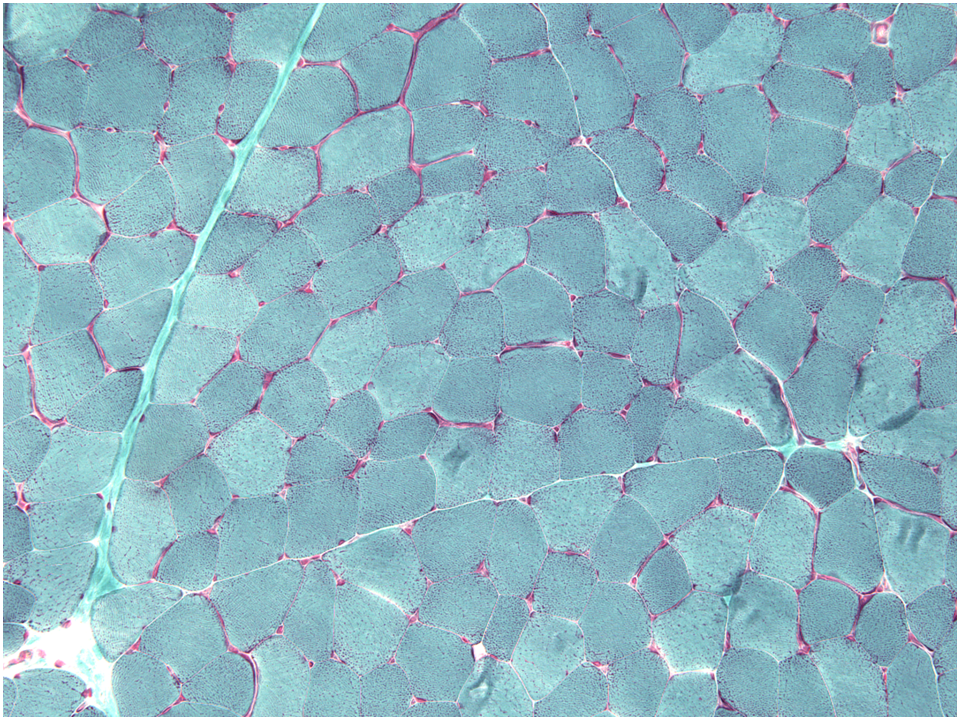

Supplement: Supplementary file 6 — FIG. S6. Right quadricep biopsy (40×) with trichrome staining showing normal intramyofibril membranes and interstitial collagen. [file ajmg0161-2040-sd6.tif]

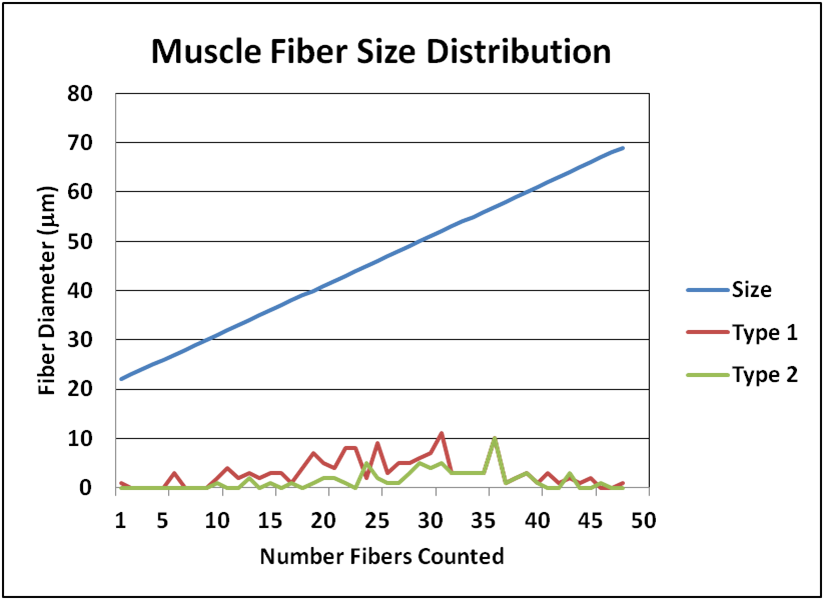

Supplement: Supplementary file 7 — FIG. S7. Distribution of size in Type 1 and Type 2 fibers in representative sections of the proband’s muscle. [file ajmg0161-2040-sd7.tif]

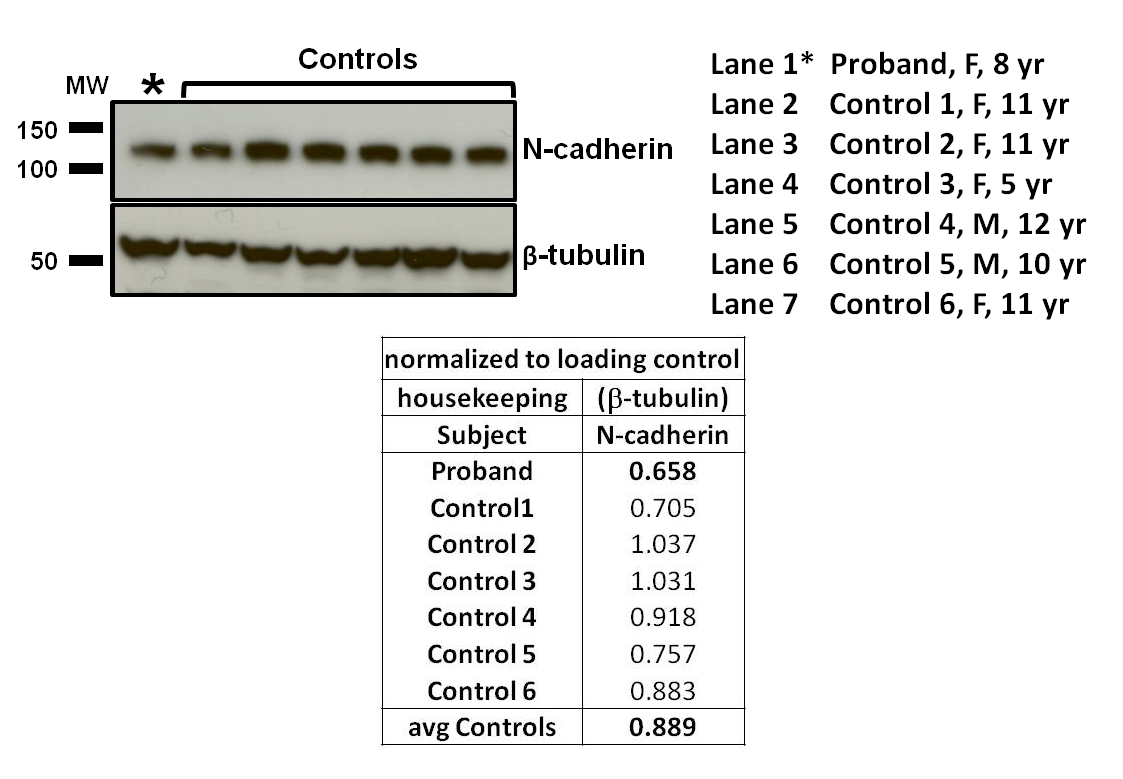

Supplement: Supplementary file 8 — FIG. S8. N-cadherin expression levels. Protein expression levels of N-cadherin in whole cell lysate from human skin fibroblasts analyzed by Western blot. b-Tubulin was used as a loading control. Values expressed are normalized to loading controls. [file ajmg0161-2040-sd8.tif]

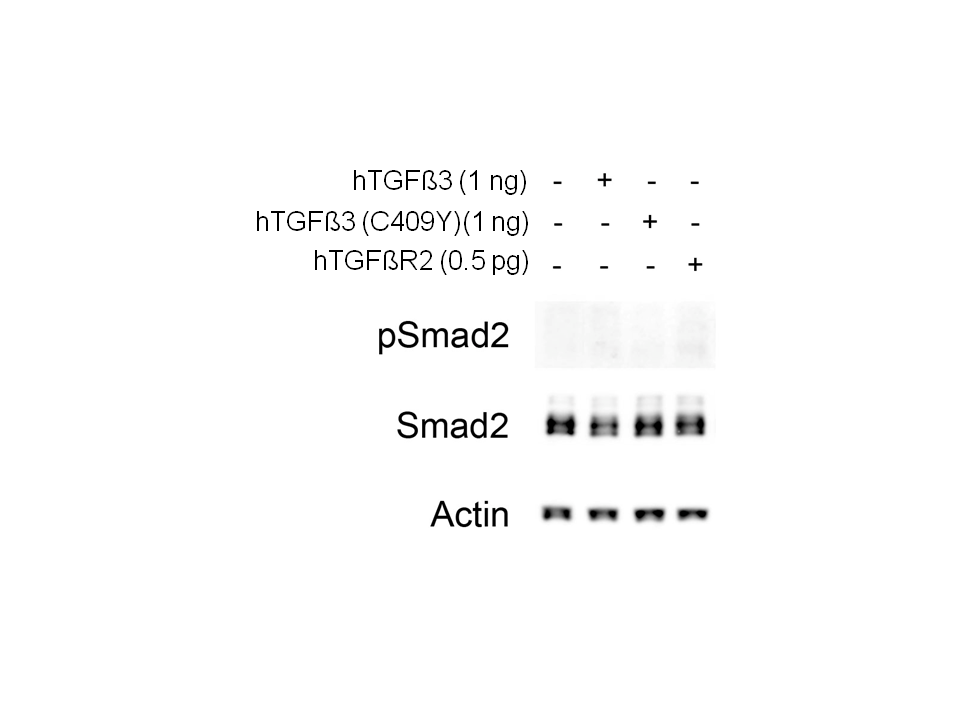

Supplement: Supplementary file 9 — FIG. S9. TGFB3 signaling in Xenopus embryos. Indicated amount of synthetic mRNAs were microinjected into Xenopus embryos after fertilization, and embryos harvested at Stage 9 forWestern blot analysis. This representative Western blot is shown to indicate that any of the injected synthetic mRNAs are not sufficient along to trigger phosphorylation of Smad2. As seen in Figure in the text, human TGFB3 and the TGFBR2 are required together to trigger the phosphorylation of Smad2. [file ajmg0161-2040-sd9.tif]
